# Internalized stigma and self-esteem among remitted patients with bipolar disorder

**DOI:** 10.1192/j.eurpsy.2022.917

**Published:** 2022-09-01

**Authors:** R. Jenhani, S. Ellouze, D. Bougacha, F. Znaidi, R. Ghachem

**Affiliations:** 1 Razi hospital, Psychiatry B, Manouba, Tunisia; 2 Hedi Chaker University Hospital, Psychiatry B, Sfax, Tunisia; 3 Razi hospital, B, Manouba, Tunisia; 4 Razi Hospital, Psychiatry B, Manouba, Tunisia

**Keywords:** bipolar disorder, self-stigma, self-esteem

## Abstract

**Introduction:**

Self-stigmatization in patients with bipolar disorder could lead to shame, self-judgement, impaired quality of life, and could negatively affect self-esteem imeding recovery.

**Objectives:**

The aim of this study was to assess self-stigma in remitted patients with bipolar disorder and to evaluate its impact on self-esteem.

**Methods:**

We conducted a cross-sectional, descriptive, and analytical study of 61 patients with bipolar disorder. Euthymia was verified using the Hamilton scale for depression and the Young scale for mania. We used the Internalized Stigma of Mental Illness (ISMI) to evaluate self-stigma, and the Rosenberg scale to assess self-esteem.

**Results:**

The mean age of patients was 43.4 years. The sex ratio was 2.4. The mean score on the ISMI was 2.36. More than half of our patients (59%) were self-stigmatized. With regard to self-esteem, the mean score obtained on the Rosenberg scale was 27.72. Low or very low self-esteem was found in 54% of patients. The most self-stigmatized patients had significantly lower self-esteem (p<10^-3^).

**Conclusions:**

Internalized stigmatization negatively affects self-esteem of patients with bipolar disorder. Psychoeducation and cognitive behavioral therapy would improve self-esteem and enhance psychosocial treatment adherence and move people with bipolar disorder toward a culture of recovery based on hope and self-determination.

**Disclosure:**

No significant relationships.

